# Compressive Neuropathy of the Facial Nerve Presenting as Bell's Palsy in a Pediatric Patient on High-Frequency Oscillatory Ventilation

**DOI:** 10.7759/cureus.8091

**Published:** 2020-05-13

**Authors:** Adebayo Adeyinka, Brisa Gulari-Jones, Keneisha Bailey-Correa, Louisdon Pierre

**Affiliations:** 1 Pediatrics, The Brooklyn Hospital Center, Brooklyn, USA; 2 Pediatrics, St. Georges University, Brooklyn, USA

**Keywords:** high-frequency, oscillatory, pediatric, bell's, ventilation, palsy

## Abstract

A three-year eight-month-old female with Werdnig Hoffman disease presented with an acute onset of respiratory failure secondary to influenza infection. The patient required conventional mechanical ventilation (CMV). Due to worsening hypoxemia on maximal support, high-frequency oscillatory ventilation (HFOV) was initiated. On recovery from her respiratory failure, she was noted to have developed a left-sided Bell's palsy. A pressure ulcer in the left mastoid area through which the facial nerve transverses was noted, with no evidence of mastoiditis. The patient fully recovered after a course of oral steroid therapy. We postulate that compression pressure might have contributed to the palsy. However, it is unclear what role the acute viral illness played in this case.

## Introduction

Bell's palsy is a unilateral acute onset paralysis of the facial nerve. The condition has been described traditionally as idiopathic but can be associated with herpes simplex viral infection. The symptoms typically peak at one week of illness and resolve over three weeks to three months period [1-3]. One common short-term complication of Bell's palsy is incomplete eyelid closure, which can result in dry eye. Another less common long-term complication is possible permanent facial weakness with muscle contractures [1-3].

Guido Werdnig and Johann Hoffmann in the 1890s presented a paper on infantile spinal muscular atrophies (SMAs). The onset usually appeared in infancy with progressive muscular weakness, floppiness, and death from aspiration pneumonia. An international consensus developed the terminology of SMA. Type 1 has an onset of symptoms before age six months. SMA type 2, starts manifesting between 6 and 18 months, and SMA type 3 starts after 18 months [4-5]. 

Werdnig Hoffman disease is an early infantile form of SMA [5]. Early-onset of symptoms is characteristic of the disease with clinical manifestations starting around six months of age. Patients afflicted are not able to sit without support, and death usually occurs around the age of two- to four years [5]. Patients with Werdnig Hoffman disease have progressive muscular weakness and are prone to developing respiratory failure secondary to an infection of the upper airway [4-5]. Respiratory support with conventional mechanical ventilation (CMV) is often required. In the event of failure of CMV, high-frequency oscillatory ventilation (HFOV) is an acceptable alternative [6-8]. The use of invasive and noninvasive mechanical ventilation has increased the survival rate of patients with SMA type 1 [9]. There are few reports of Bell's palsy associated with the compression of one side of the face. In our patient, we postulate two etiologies; prolonged compression of the left side of the face and a sequela of the influenza infection.

## Case presentation

**Figure 1:**
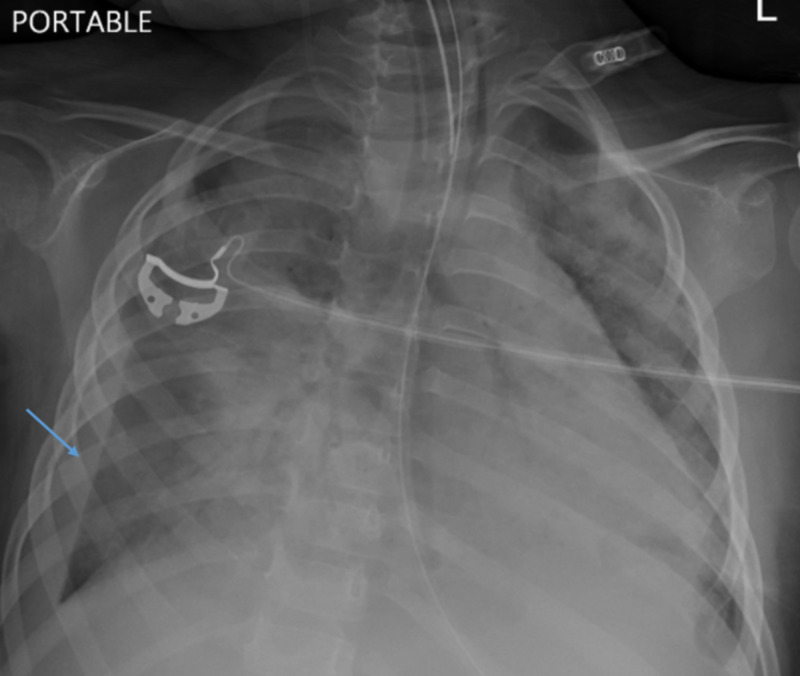
Chest X-ray demonstrating extensive bilateral lung infiltrates and a right pleural effusion.

Our patient is a three-year and eight-month-old female with Werdnig Hoffman disease and a seizure disorder. Two days after testing positive for influenza B infection, the patient presented to the pediatric ER with respiratory distress. On physical examination, vital signs were significant for tachycardia, fever (103.9°F) with oxygen saturation below 80% on room air. The patient was tachypneic with subcostal and supra-sternal retractions. The patient remained cyanotic on facemask oxygen with poor air entry on auscultation. The oxygen saturation increased to the low 90s on a nonrebreather mask. Her labs were significant for bandemia, leukopenia, thrombocytopenia, hypokalemia, and hypocalcemia. She was subsequently intubated and started on synchronized intermittent mandatory ventilation (SIMV) volume control (VC), subsequently changed to pressure regulated volume control (PRVC) because of worsening hypoxemia. A chest X-ray revealed extensive bilateral lung infiltrates and the suspicion of a pleural effusion (Figure 1). 

**Figure 2:**
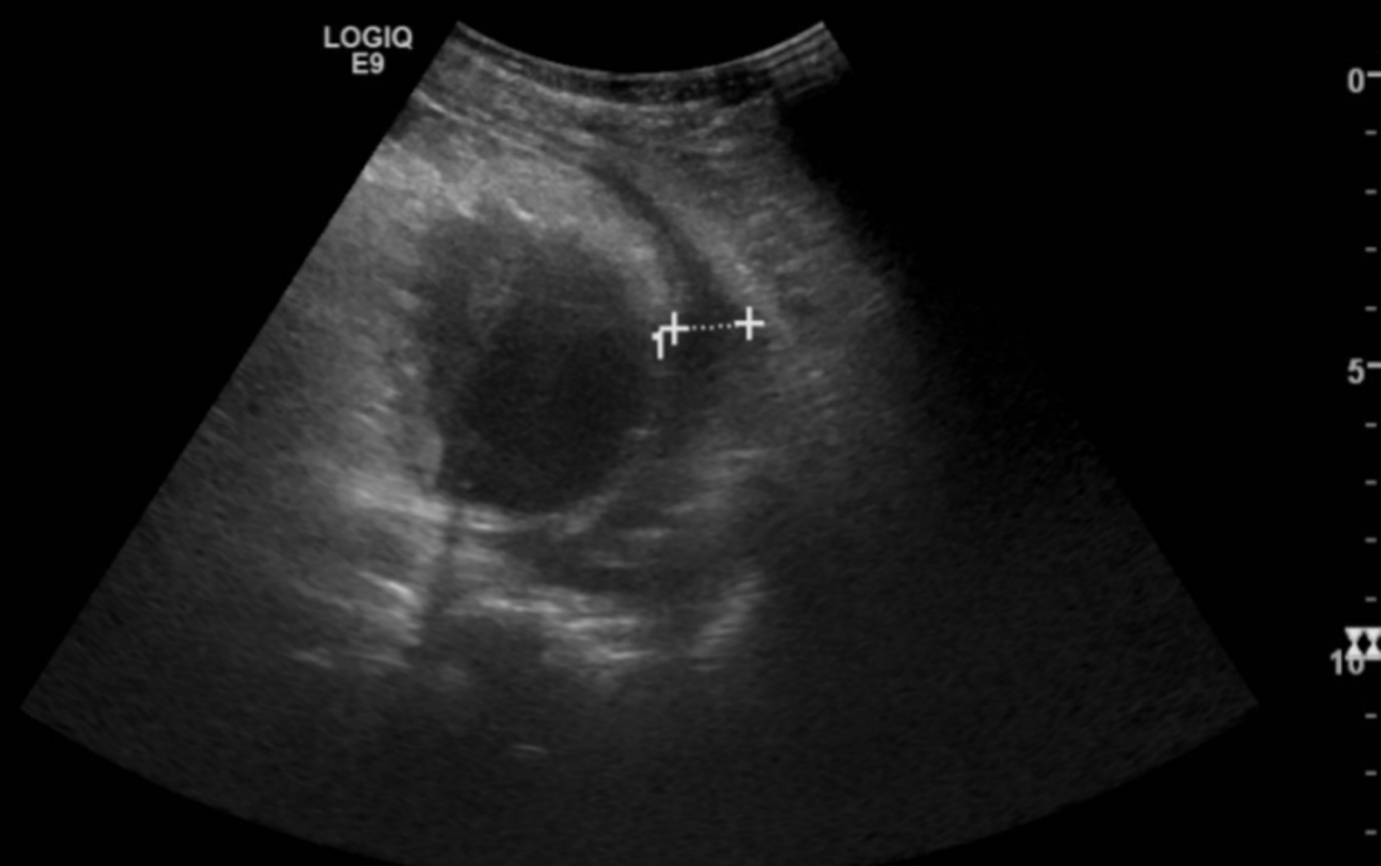
Chest ultrasound showing moderate to large pleural effusion.

A chest ultrasound confirmed the presence of a moderate to large right pleural effusion (Figure 2).

**Figure 3:**
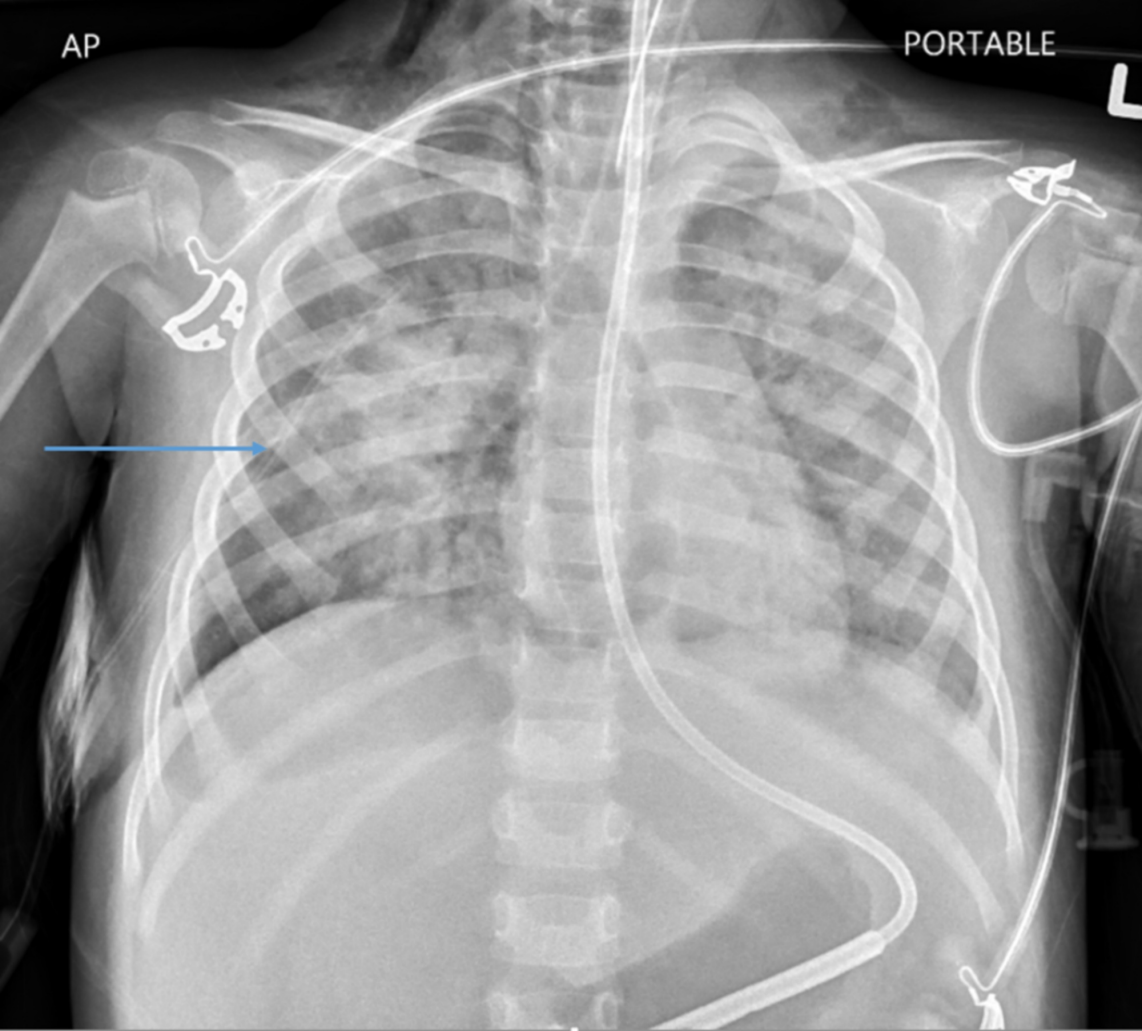
Chest X-ray showing the thoracostomy tube.

A thoracostomy tube was placed on the right side, which drained 120 mL of serosanguineous fluid (Figure 3). 

On the second day of hospitalization, the patient was noted to have persistent hypoxemia on PRVC and was placed on HFOV. Antimicrobial coverage was initiated with ceftriaxone and vancomycin. To broaden the coverage, ceftriaxone was replaced with cefepime on hospital day 3. Blood cultures, urine cultures, and tracheal aspirates were all negative. On hospital day 8, due to the improvement of her respiratory failure, the patient transitioned back to SIMV. On hospital day 14, a spontaneous breathing trial with continuous positive airway pressure (CPAP) with pressure support (PS) was successful, and the patient extubated to high flow nasal cannula.

On day 16, the patient exhibited a deviation of her smile towards the right with failure to close her left eye completely. This correlated with a grade 3 left facial nerve Bell's palsy. Of note, the patient had developed a pressure ulcer on her left mastoid process secondary to lying on her left side during HFOV. The patient was started on 30 mg PO prednisone daily, with some mild improvement in motor function by the time of discharge.

## Discussion

**Figure 4:**
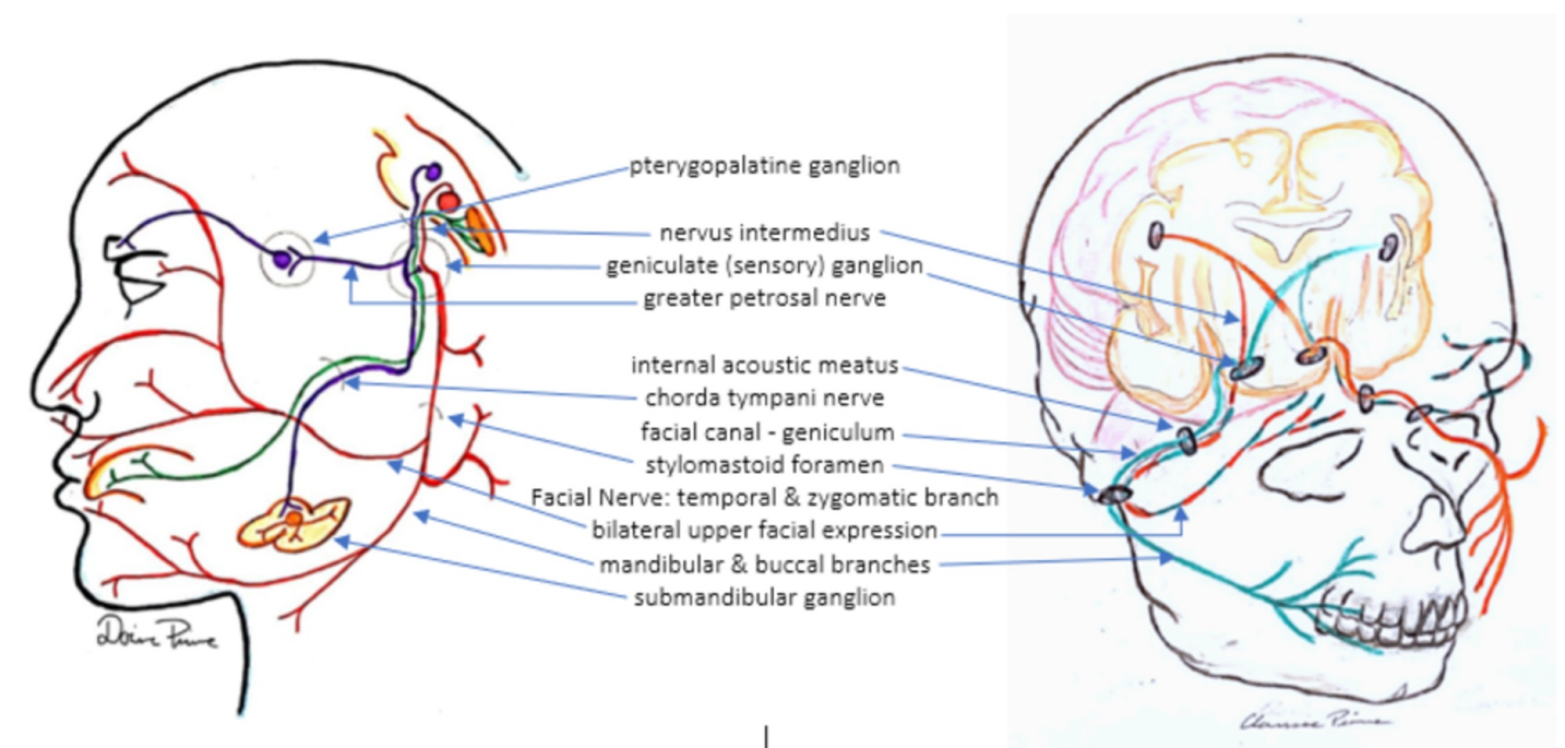
Diagram depicting the course of the facial nerve. Adapted from Facial nerve. In: Wikipedia [Internet]. 2020 [cited April 24, 2020]. Available from: https://en.wikipedia.org/w/index.php?title=Facial_nerve&oldid=951988696

Our patient was of an age where she may have been particularly vulnerable to developing Bell's palsy, as she was three years and eight months. The anatomy and course of the facial nerve from birth until four years of age lend vulnerability to injury as it lies just beneath the skin as it exits the temporal bone. The facial nerve originates at the pontomedullary junction from two nerve roots. The motor root innervates muscle fibers of the second branchial arch mesoderm. The sensory fibers of the nervus intermedius supply the palate, tongue. The preganglionic parasympathetic fibers terminate in the pterygopalatine and submandibular ganglia (Figure 4).

The roots merge into the internal acoustic meatus. At the lateral end of the meatus, the facial nerve goes into the facial canal. The nerve then takes a sharp turn posteriorly at the geniculum. The geniculate (sensory) ganglion and the greater petrosal nerve to the pterygopalatine ganglion arise from this geniculum. The nerve continues in the facial canal posteriorly along the medial wall of the tympanic cavity, coursing above the fenestra vestibuli and arching downward and laterally to emerge from the stylomastoid foramen just after giving off the chorda tympani nerve [10-11]. 

The patient developed a pressure ulcer on the left mastoid region while on HFOV. A few days later, ipsilateral Bell's palsy developed. There are some reports of Bell's palsy after prolonged compression on one side of the face. Usukura et al. described this finding in a 70-year-old woman who underwent a right total hip arthroplasty, which lasted for nine and a half hours during which the patient laid on her left side for the duration of the procedure [12]. The symptoms developed on the next postoperative day compared to a subacute presentation in our patient, manifesting on day 16 of illness. A prolonged period of mechanical ventilation, sedation, and neuromuscular blockade may have contributed to this late discovery.

Acute otitis media is the most common etiology of an acute onset of facial nerve palsy in children. Acute infection with influenza B is relevant in our case because this virus is frequently associated with the development of Bell's palsy [13]. Our patient remained on HFOV for seven days, with most time spent in the left lateral decubitus position. Interestingly, there have been reports of newborns who were started on CPAP and subsequently developed Bell's palsy [14]. Both were born premature and treated with nasal CPAP for 15 days, and the other for seven days before developing facial palsy. Compression of the seventh cranial nerve potentially contributed to Bell's palsy in these patients as well as our patient [14].

Melkersson-Rosenthal syndrome is a rare cause of facial nerve paralysis characterized by persistent or recurrent orofacial edema, relapsing peripheral facial paralysis, and furrowed tongue. There is a report of a 45-year-old woman with Melkersson-Rosenthal syndrome who developed respiratory failure with ARDS. In this instance, the cause of facial paralysis was related to an underlying syndrome [15]. This syndrome has also been reported in children [16-17]. The administration of corticosteroids as a treatment for Bell's palsy has shown mixed results. The majority of children with this disorder have a complete recovery with or without the use of steroids [18-20]. Our patient displayed mild improvement within a few days after the initial presentation of the palsy. After a tapered course of prednisone, the patient had a complete recovery of the facial nerve function. 

## Conclusions

In summary, there are only a few documented instances of the development of Bell's palsy in patients following sedation and the use of mechanical ventilation, suggesting compressive injury to the facial nerve. Infectious, traumatic, or compressive injury are known precipitants of Bell's palsy in children. Our patient had a congenital lower motor neuron disorder, presenting with an acute viral infection and also required mechanical ventilation with HFOV. Clear differentiation between a compressive and an infectious etiology is somewhat difficult to tease out in this case. Nonetheless, we recommend vigilance for the proper positioning of patients who are nonmobile and paralyzed during their treatment in the ICU. 
